# Anticancer Chemotherapy-Induced Atherosclerotic Cardiovascular Disease: A Comprehensive Review

**DOI:** 10.3390/life15020245

**Published:** 2025-02-06

**Authors:** Juan S. Izquierdo-Condoy, Marlon Arias-Intriago, Diego Alexander Becerra Cardona, Susana García-Cañarte, Paul Vinueza-Moreano

**Affiliations:** 1 One Health Research Group, Universidad de las Américas, Quito 170124, Ecuador; 2 Facultad Ciencias de la Salud, Universidad del Quindío, Armenia 630001, Colombia; 3 Facultad de Ciencias Médicas, Universidad de Buenos Aires, Buenos Aires C1121ABG, Argentina; 4 División de Cardiología, Hospital General Jose María Ramos Mejia, Buenos Aires C1221ADC, Argentina

**Keywords:** cardio-oncology, atherosclerosis, anticancer agents, cardiovascular risk, oxidative stress, endothelial dysfunction

## Abstract

The introduction of anticancer agents has transformed oncology, significantly improving survival rates. However, these therapies have introduced unintended cardiovascular risks, with atherosclerovascular disease (ASCVD) emerging as a leading cause of morbidity and mortality among cancer survivors. The development of ASCVD in this population involves multifactorial mechanisms, including endothelial dysfunction, oxidative stress, systemic inflammation, and disrupted lipid metabolism. This review examines the various mechanisms through which anticancer chemotherapy contributes to ASCVD and highlights strategies for risk assessment and management. Each class of anticancer agents presents distinct cardiovascular challenges: anthracyclines induce oxidative stress and endothelial damage, promoting foam cell formation and plaque progression; taxanes and vascular endothelial growth factor (VEGF) inhibitors impair lipid metabolism and vascular stability; anti-metabolites exacerbate endothelial injury through reactive oxygen species; and mTOR inhibitors, hormonal therapies, tyrosine kinase inhibitors, and immune checkpoint inhibitors disrupt lipid profiles and inflammatory pathways, increasing the risk of plaque rupture and thrombosis. Mitigating chemotherapy-induced ASCVD necessitates a comprehensive, multidisciplinary approach. Detailed pre-treatment cardiovascular risk assessments must address traditional and cancer-specific risk factors, including demographics, pre-existing conditions, and modifiable behaviors such as smoking and inactivity. Pharmacological interventions like statins and angiotensin-converting enzyme (ACE) inhibitors, paired with lifestyle modifications, are essential to reducing ASCVD risk. In resource-limited settings, cost-effective strategies should be prioritized to enhance accessibility. Establishing cardio-oncology units facilitates care coordination, while long-term surveillance enables timely detection and intervention. These strategies collectively improve cardiovascular outcomes and survivorship in diverse patient populations.

## 1. Introduction

Advances in cancer treatment have significantly improved survival rates and outcomes for patients with malignancies [1]. However, these therapies are not without risks, as many are associated with adverse cardiovascular effects, including cardiotoxicity, heart disease, and other cardiovascular disorders [2,3,4]. Among these complications, long-term cardiovascular damage is particularly concerning for cancer survivors, with atherosclerovascular disease (ASCVD)—a chronic inflammatory condition of the arterial wall—emerging as a critical complication of cancer therapy [5,6].

Research has shown that cancer survivors face a heightened risk of ASCVD, driven by prolonged survival and cumulative exposure to cytotoxic therapies. ASCVD has now become a leading cause of morbidity and mortality in this population [7,8,9]. While cancer and ASCVD share common risk factors such as age, tobacco use, and diabetes, the association between cancer and ASCVD persists even after adjusting for these factors [6]. Individuals with a history of cancer have been found to have a 3.42-fold higher likelihood of an elevated ASCVD risk index [9]. Furthermore, chemotherapy exacerbates this risk; in a longitudinal study of 1413 breast cancer patients with ASCVD, those receiving chemotherapy demonstrated a 1.7-fold higher risk of mortality from ASCVD compared to patients not undergoing chemotherapy [10,11].

Certain chemotherapeutic agents, particularly anthracyclines, are strongly implicated in accelerating the development of atherosclerosis and its complications [12]. The mechanisms underlying chemotherapy-induced atherosclerosis are multifactorial, involving endothelial damage, arterial thrombosis, impaired microcirculation, and long-term disruptions in lipid metabolism [3,13]. These vascular changes can culminate in coronary atherosclerotic heart disease, characterized by the formation of atherosclerotic lesions that lead to coronary artery stenosis or obstruction, ultimately resulting in myocardial ischemia, hypoxia, or necrosis [6,14].

This review seeks to provide a comprehensive overview of the mechanisms and risks associated with chemotherapy-induced atherosclerosis and to offer insights into clinical management strategies. By elucidating the interplay between oncology and cardiovascular medicine, this work aims to contribute to the development of strategies that mitigate cardiovascular risks while preserving the therapeutic efficacy of cancer treatments.

## 2. Materials and Methods

### 2.1. Research Question

This comprehensive review aimed to identify and synthesize evidence on the mechanisms by which cancer chemotherapy contributes to the development of ASCVD and to explore strategies for risk assessment and management.

### 2.2. Study Design

A narrative literature review was conducted to capture the breadth of available evidence on the relationship between anticancer chemotherapeutic agents and the development of ASCVD. A wide range of sources were included, such as original research articles, clinical trials, cohort studies, case series, case reports, literature reviews, systematic reviews, and meta-analyses. Incorporating diverse study types allowed for a comprehensive understanding of the multifactorial pathways linking cancer chemotherapy to ASCVD.

### 2.3. Search Strategies

A systematic search of the literature in English and Spanish was conducted using major scientific databases, including PubMed/Medline, SCOPUS, Web of Science, and Google Scholar. References of key articles were also manually searched to ensure comprehensive coverage. No restrictions on publication date were applied to capture both historical insights and recent advancements.

The following keywords were employed in the title or abstract to identify relevant studies: “cancer”, “chemotherapy”, “atherosclerosis”, “endothelial dysfunction”, “oxidative stress”, “immune checkpoint inhibitors”, “tyrosine kinase inhibitors”, and “cardio-oncology”. Boolean operators (AND, OR) were used to refine search strategies, optimizing the retrieval of relevant literature.

### 2.4. Selection Criteria

The review included studies focusing on the effects of cancer chemotherapy on cardiovascular health, with a particular emphasis on mechanisms contributing to ASCVD. Only evidence derived from human studies, including clinical trials, observational studies, and case series, was considered. Furthermore, studies exploring therapeutic or preventive strategies to address chemotherapy-induced ASCVD were also included to provide a comprehensive perspective on risk management.

Conversely, studies conducted exclusively on animal models or in vitro experiments were excluded, as were those primarily addressing non-atherosclerotic cardiovascular complications of chemotherapy, such as arrhythmia or cardiomyopathy. Additionally, studies reporting outcomes unrelated to cardiovascular health or failing to address the mechanisms of ASCVD development were deemed ineligible for inclusion.

### 2.5. Bias Assessment

To minimize bias, data extraction was independently performed by two authors (i.e., JSI-C and MAI) at separate times. Any discrepancies or disagreements were resolved through discussion with the entire research team. This multi-author approach enhanced the reliability and validity of the data collection process by incorporating diverse perspectives.

### 2.6. Data Synthesis

All included studies were reviewed to extract data on the mechanisms by which anticancer chemotherapy contributes to ASCVD. Key mechanisms investigated included endothelial dysfunction, oxidative stress, systemic inflammation, and disrupted lipid metabolism. Data on pharmacological and non-pharmacological strategies to mitigate the risk of ASCVD in cancer survivors were also extracted.

The extracted data were systematically categorized according to chemotherapy class (e.g., anthracyclines, taxanes, vascular endothelial growth factor inhibitors, immune checkpoint inhibitors) and analyzed to assess relevance to the review objectives. A multidisciplinary framework was applied, integrating findings from oncology, cardiology, and pharmacology to provide a comprehensive evaluation of mechanisms, risks, and management strategies for chemotherapy-induced ASCVD.

## 3. Mechanisms of Atherosclerosis Induction by Chemotherapy

Anticancer agents contribute to atherosclerosis through various mechanisms, including the following:

### 3.1. Dyslipidemia

Chemotherapeutic agents disrupt lipid metabolism, promoting the accumulation of lipids in arterial walls and accelerating atherosclerotic plaque formation. Alterations in plasma lipid composition, such as elevated low-density lipoproteins (LDLs) and reduced high-density lipoproteins (HDLs), exacerbate atherosclerosis progression [15]. Tyrosine kinase inhibitors (TKIs) and angiogenesis inhibitors are particularly implicated, as they increase oxidized LDL (oxLDL), a critical factor in plaque formation. For instance, nilotinib has been associated with significant dyslipidemia and a heightened risk of peripheral artery disease.

### 3.2. Inflammation

Chronic inflammation is a key driver of chemotherapy-induced atherogenesis. Agents such as VEGF inhibitors (e.g., bevacizumab) and immunotherapies alter the balance of inflammatory cytokines, inducing the release of tumor necrosis factor-alpha (TNF-α), interleukins (IL-6, IL-1β), and interferon-gamma (IFN-γ). These pro-inflammatory molecules facilitate monocyte adhesion to the vascular endothelium and their differentiation into macrophages, leading to foam cell formation, a hallmark of atherosclerotic plaques [16]. The persistent inflammatory state is mediated by the secretion of the senescence-associated secretory phenotype (SASP), further attracting immune cells and exacerbating vascular damage [17].

### 3.3. Oxidative Stress

Chemotherapy generates reactive oxygen species (ROS), which directly damage the vascular endothelium and oxLDL—a critical initial step in atherogenesis [11]. ROS also induce apoptosis in endothelial and vascular smooth muscle cells, destabilizing plaques and increasing the risk of rupture. Furthermore, ROS-mediated DNA damage, including telomeric DNA, triggers the DNA damage response (DDR), amplifying oxidative stress and promoting plaque formation [18].

Oxidative stress is a well-documented consequence of cancer chemotherapy; however, the specific mechanisms underlying its generation vary according to the type of chemotherapeutic agent used. Thus, oxidative stress can be posited as a key process in the pathogenesis of atherosclerosis, contributing to the development of atheromatous plaques in cancer patients undergoing treatment. For example, antihormonal therapies have been shown to increase cholesterol synthesis, leading to lipid accumulation and inflammation, which, in turn, promotes immune cell recruitment and ROS production. On the other hand, taxanes exert their effects by disrupting cell division and inducing mitotic arrest, leading to the accumulation of ROS as a byproduct of cellular stress [18].

### 3.4. Endothelial Dysfunction

Endothelial dysfunction, characterized by reduced nitric oxide (NO) bioavailability and increased endothelin signaling, is a pivotal mechanism in chemotherapy-induced atherosclerosis [19]. Angiogenesis inhibitors like bevacizumab reduce NO and prostacyclin (PGI2) production, essential for vasodilation and endothelial homeostasis. This results in vasoconstriction, capillary rarefaction, and vascular smooth muscle proliferation, leading to vascular narrowing, platelet activation, and thrombus formation [16]. The hypoxia induced by prolonged VEGF inhibition further exacerbates atherosclerosis progression and chronic hypertension. Reduced NO bioavailability also increases leukocyte adhesion and vascular permeability, compounding vascular injury and the risk of ischemic events [20].

**Figure 1:**
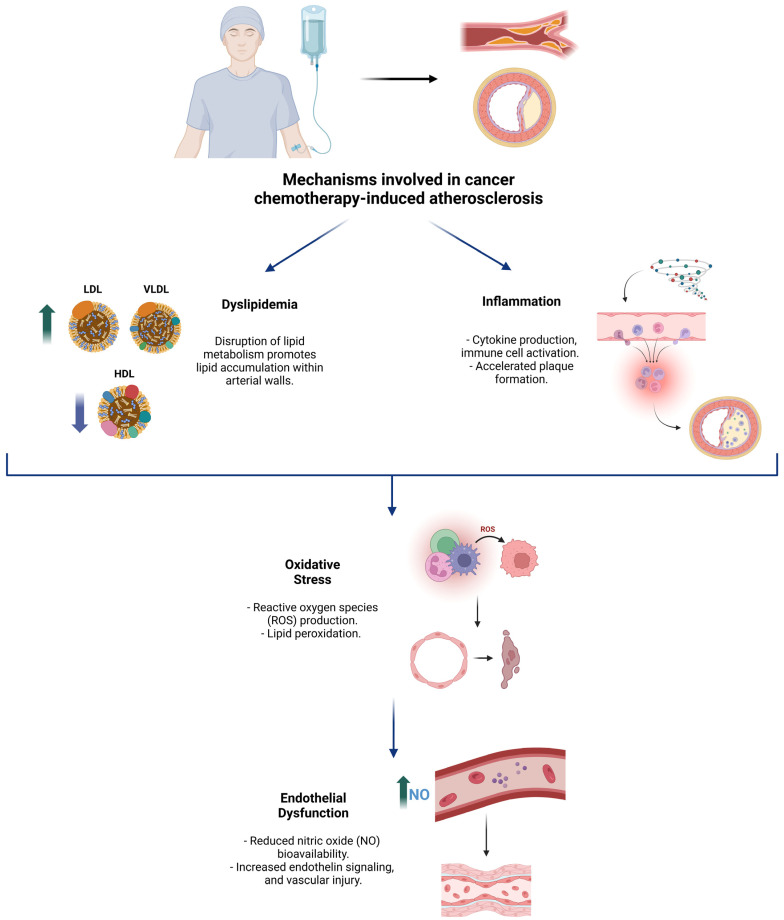
Overview of the key pathophysiological mechanisms underlying cancer chemotherapy-induced atherosclerosis.

Figure 1 illustrates the main mechanisms associated with chemotherapeutic agent-induced atherosclerotic disease, highlighting their interrelated pathways, including endothelial dysfunction, oxidative stress, systemic inflammation, and lipid metabolism alterations.

## 4. Pharmacological Groups Related to Atherosclerosis and Their Effects

### 4.1. Anthracyclines

Anthracyclines, such as doxorubicin, are widely used chemotherapy agents that exert their effects through several mechanisms, including inhibition of topoisomerase II, induction of mitochondrial dysfunction, and generation of ROS [21,22]. These processes inhibit DNA and RNA synthesis, impair endothelial function, and elevate oxidative stress levels. In addition, anthracyclines interfere with cholesterol efflux by disrupting the activity of upstream regulators, such as liver X receptor-alpha (LXR-α) and peroxisome proliferator-activated receptor-gamma (PPAR-γ), which are critical in regulating transporters like ATP-binding cassette transporter A1 (ABCA1) [21,22]. These disruptions create a complex interplay between metabolic pathways and the immune system, influencing inflammation and immunity [15,16,23].

Anthracyclines also increase apolipoprotein B levels, contributing to the accumulation of low-density lipoproteins (LDLs) in the intimal layer of blood vessels, a critical step in atherosclerosis. Although anthracyclines reduce the activity of 3-hydroxy-3-methylglutaryl-coenzyme A reductase (HMGCR)—which theoretically could lower LDL levels—this paradoxically promotes foam cell formation, a hallmark of atherosclerotic plaque development [15].

Furthermore, oxidative stress induced by anthracyclines damages endothelial cells, leading to apoptosis and exposure of the underlying vascular wall [24]. This creates a pro-thrombotic environment, further accelerating the progression of atherosclerosis. Preclinical studies have also demonstrated that anthracyclines may promote vascular smooth muscle cell proliferation, contributing to both plaque formation and instability [25,26].

### 4.2. Taxanes

Taxanes, including paclitaxel and docetaxel, exert their anticancer effects by stabilizing GDP-bound tubulin in microtubules, thereby disrupting their dynamic assembly and preventing effective microtubule function during cell division [27]. These versatile drugs are widely used in the treatment of various solid tumors and are a cornerstone of breast cancer therapy [15].

In a study involving 80 women with breast cancer, a single infusion of paclitaxel was shown to influence the expression of at least 188 proteins. Proteomic analysis revealed that many of these proteins are critical in lipid metabolism, particularly those related to lipoprotein pathways. Sharma et al. demonstrated that paclitaxel induces increased activity of 3-hydroxy-3-methylglutaryl-coenzyme A reductase (HMGCR) in human hepatocytes, a key enzyme in cholesterol biosynthesis. Additionally, paclitaxel was found to reduce apolipoprotein B activity and LDL receptor expression, both of which play essential roles in the clearance of LDL from the bloodstream [15,26,28].

### 4.3. VEGF Inhibitors

The vascular endothelial growth factor (VEGF) signaling pathway plays a critical role in the progression of various malignancies by promoting tumor angiogenesis. VEGF inhibitors, such as bevacizumab, sorafenib, and axitinib, are widely used in the treatment of colon cancer, lung cancer, glioblastoma, and renal cell carcinoma [29]. While effective in targeting tumor angiogenesis, these agents also disrupt endothelial homeostasis, leading to cardiovascular complications [16,26].

VEGF inhibitors impair vasodilation by reducing the bioavailability of NO and prostacyclin (PGI2), two key mediators of vascular relaxation. This disruption promotes vasoconstriction and increases vascular resistance [30]. Additionally, these agents enhance platelet activation and aggregation, which predisposes patients to microvascular thrombosis [15,26].

Chronic endothelial dysfunction induced by VEGF inhibitors fosters a pro-inflammatory environment, activating macrophages and vascular smooth muscle cells [31]. This process accelerates the formation and progression of lipid-rich atherosclerotic plaques. Furthermore, clinical data indicate that patients treated with VEGF inhibitors have a higher incidence of hypertension, a well-established independent risk factor for the progression of atherosclerosis [15].

### 4.4. Anti-Metabolites

Anti-metabolites, including 5-fluorouracil (5-FU) and capecitabine, are purine and pyrimidine analogs that interfere with cell proliferation by incorporating into DNA. This diverse group of medications is widely used to treat various malignancies [15,16].

In preclinical studies, 5-FU has been associated with cholesterol-lowering effects, as observed in a rabbit model [32]. However, this link has yet to be established in humans. Methotrexate, another anti-metabolite, has been shown to decrease cholesterol levels by altering the expression of ABCA1 and 27-hydroxylase enzymes [33]. Despite these findings, the Cardiovascular Inflammation Reduction Trial demonstrated that low-dose methotrexate minimally reduced LDL, triglyceride (TG), and HDL levels compared to placebo, with no observed cardiovascular benefit [15,26].

Anti-metabolites also contribute to endothelial dysfunction through multiple mechanisms. Endothelial stress induced by these agents upregulates pro-inflammatory cytokines and adhesion molecules, such as ICAM-1 and VCAM-1, which promote monocyte adhesion and migration into the arterial intima [33]. Concurrently, anti-metabolites increase ROS levels, deplete antioxidant reserves (e.g., glutathione), and induce mitochondrial dysfunction [34]. These effects exacerbate endothelial injury, creating a pro-inflammatory environment that destabilizes plaques and enhances their vulnerability to rupture [16].

Clinically, patients undergoing anti-metabolite therapy often report chest pain, which is hypothesized to result from coronary vasospasm—an early indicator of vascular compromise. While some anti-metabolites have no known association with dyslipidemia, the overall impact of this drug class on ASCVD risk remains uncertain [16].

### 4.5. mTOR Inhibitors

mTOR inhibitors, such as everolimus and sirolimus, function by inhibiting the mammalian target of rapamycin (mTOR) signaling pathway, which regulates gene transcription and protein synthesis related to cell proliferation and immune cell differentiation [35]. While these agents are primarily used as immunosuppressive therapies to prevent organ rejection, they are also utilized as targeted anti-neoplastic agents in certain cancers [15,16,26].

In addition to their effects on cell proliferation, mTOR inhibitors influence lipid metabolism by downregulating lipogenic enzymes and reducing LDL receptor activity. This results in dyslipidemia, characterized by elevated LDL cholesterol and triglycerides [36]. Beyond these lipid alterations, mTOR inhibitors suppress endothelial cell proliferation and impair vascular repair mechanisms, compromising the integrity of the endothelial barrier. This disruption facilitates the infiltration of lipids and inflammatory cells into the vascular wall, further contributing to the pathogenesis of atherosclerosis [15].

Notably, mTOR inhibitors also impair autophagy, a critical cellular process for maintaining endothelial homeostasis [37]. Impaired autophagy exacerbates oxidative stress and promotes plaque instability, increasing the risk of atherosclerotic events [16].

Despite these concerns, the clinical significance of mTOR inhibitor-induced dyslipidemia remains unclear, as mTOR inhibition may also suppress pathways that contribute to atherosclerosis progression. Further research is needed to fully understand the net cardiovascular effects of mTOR inhibitors and their role in atherosclerosis development [16].

### 4.6. Hormonal Therapies

Estrogen and testosterone play critical roles in regulating cholesterol synthesis, transport, and metabolism. Estrogen inhibits liver HMG-CoA reductase (HMGCR), resulting in decreased cholesterol synthesis. Conversely, testosterone deficiency has been shown to reduce the expression of nuclear receptors such as liver X receptor (LXR) and peroxisome proliferator-activated receptor-gamma (PPAR-γ), both of which are essential for maintaining serum cholesterol levels [15,16,26,28].

Hormonal therapies targeting estrogen and testosterone, including androgen deprivation therapy (ADT) and selective estrogen receptor modulators (SERMs), are standard treatments for prostate and breast cancers, respectively. These therapies significantly alter systemic metabolic profiles [38]. ADT reduces androgens, which normally exert atheroprotective effects by stimulating NO production and inhibiting platelet aggregation. Consequently, ADT increases the risk of cardiovascular events; longitudinal studies have shown that prostate cancer patients receiving ADT have approximately a 20% higher risk of cardiovascular events compared to those not undergoing hormone therapy [15,26].

SERMs, such as tamoxifen, offer protective effects against ASCVD and dyslipidemia by lowering LDL cholesterol levels. However, in certain cases, SERMs may increase triglyceride levels, contributing to lipid accumulation in the arterial wall and plaque formation. For instance, a study of 141 breast cancer patients treated with SERMs for three years reported that lipid profile alterations normalized within six months of discontinuing therapy [39]. Notably, while some studies have observed elevations in TG levels following SERM treatment [40,41], others have not [42].

Aromatase inhibitors (AIs), which reduce circulating estrogen by inhibiting its production, are associated with higher cardiovascular risks compared to SERMs [43,44]. Data suggest that AIs increase dyslipidemia at least three months after therapy cessation and are linked to a 1.18-fold higher risk of cardiovascular events compared to placebo [45]. However, a lack of direct cardiovascular outcome comparisons between AIs and placebo complicates the assessment of their full impact. The apparent cardiovascular risk of AIs may also be influenced by comparisons to the protective effects of SERMs [46].

Chemotherapy-induced ovarian failure further compounds cardiovascular risks in cancer survivors. Ovarian follicles, due to their high growth rates, are highly sensitive to chemotherapy, leading to a loss of estrogen production and premature ovarian failure. This estrogen deficiency fosters metabolic syndrome, characterized by increased LDL cholesterol, triglycerides, and insulin resistance, which independently contribute to ASCVD risk [15].

### 4.7. Tyrosine Kinase Inhibitors (TKIs)

Tyrosine kinase inhibitors (TKIs), including imatinib, nilotinib, and ponatinib, are essential in the treatment of gastrointestinal, genitourinary, hematologic, and lung cancers. In addition to their on-target anticancer effects, TKIs exert off-target effects by inhibiting various kinases involved in pathways critical for tumor angiogenesis [47]. This inhibition impairs endothelial repair and reduces NO production. Furthermore, TKIs increase oxidative stress by promoting ROS production and lipid peroxidation, contributing to endothelial dysfunction [16].

Imatinib, a first-generation TKI targeting BCR-ABL1, has demonstrated unique effects on lipid metabolism. It reduces the cytoplasmic phosphorylation of LDL receptor-related protein, a key component of LDL signaling that influences lysosomal enzyme activation, glucose-induced insulin secretion, and cholesterol metabolism [15].

Preclinical studies have highlighted imatinib’s protective effects on lipid metabolism and cardiovascular health. In a rabbit model fed a high-cholesterol diet, imatinib therapy reduced cholesterol levels and mitigated the toxic effects of hyperlipidemia on the aorta and liver [48]. These effects were mediated by decreases in lipid levels, C-reactive protein, and hepatic enzymes, indicating that imatinib acts through multiple pathways to counter atherosclerosis and vascular toxicity [49]. These findings suggest that imatinib may have a protective role in lipid metabolism, in contrast to other TKIs that may exert opposing or synergistic effects depending on their target receptors [15].

The lipid-modulating effects of newer-generation TKIs, which are increasingly used in the treatment of solid tumors such as gastrointestinal, genitourinary, and lung malignancies, remain an area of active investigation [50]. Emerging data suggest a paradoxical relationship between increased lipid levels and improved overall survival in cancer patients treated with novel TKIs [50]. While this phenomenon requires further validation, it underscores the importance of lipid metabolism as a factor influencing mortality risk in TKI recipients. However, lipid metabolism is likely only one of several factors determining outcomes in these patients [15].

### 4.8. Alkylating Agents

Alkylating agents exert their anticancer effects by cross-linking strands of DNA and RNA, thereby preventing cell division [51]. However, their association with dyslipidemia remains unclear. In an in vitro study, Sharma et al. demonstrated that exposing human hepatocytes to cyclophosphamide did not significantly impact metabolic pathways involved in lipogenesis [16].

Findings from animal models provide mixed results. Cyclophosphamide induced dyslipidemia in rats at toxic doses, suggesting dose-dependent adverse effects [52]. Conversely, low-dose cyclophosphamide treatment was associated with reduced progression of atherosclerotic disease in a mouse model, highlighting potential cardioprotective effects at lower dosages [53].

Clinical data on humans are similarly variable. Cyclophosphamide regimens that do not include anthracyclines appear to have a neutral or even beneficial impact on lipid profiles, with some studies reporting improved LDL cholesterol levels. However, data on dyslipidemia associated with other alkylating agents remain limited, likely due to their restricted clinical use [26,28].

### 4.9. Platinum-Based Agents

Platinum-based compounds, such as cisplatin, are positively charged platinum ions surrounded by negatively charged anions. These agents cross-link DNA, inhibiting transcription and disrupting mRNA translation, ultimately inducing cell death [16,26].

Beyond their cytotoxic effects, platinum agents contribute to a pro-inflammatory and pro-thrombotic state by increasing the production of pro-inflammatory cytokines, fibrinogen, and von Willebrand factor. They also impair endothelial function by reducing NO production, which disrupts vascular relaxation. This endothelial dysfunction facilitates the infiltration of lipids and immune cells into the arterial wall, promoting vascular inflammation and atherosclerotic plaque development [26].

While cholesterol metabolism appears to influence the efficacy of platinum compounds, evidence linking these agents to long-term changes in cholesterol levels is limited. However, clinical studies have shown that adjuvant chemotherapy regimens primarily consisting of cisplatin, carboplatin, and nedaplatin are associated with increases in total cholesterol, LDL, HDL, and triglycerides at the end of the treatment period [15,54].

### 4.10. Immune Checkpoint Inhibitors (ICIs)

Immune checkpoint inhibitors (ICIs), such as anti-PD-1 and anti-CTLA4 antibodies, enhance anti-tumor immune responses by blocking inhibitory pathways that suppress T-cell activation [55]. While effective in combating cancer, these therapies disrupt immune homeostasis in the arterial wall, leading to increased T-cell activation and cytokine production, which accelerate inflammation within atherosclerotic plaques [56].

The widespread adoption of ICIs and stem cell transplantation has significantly altered treatment regimens for many common cancers. However, these modalities have been associated with an increased risk of ASCVD [26].

Animal studies have demonstrated that PD-1 exerts anti-inflammatory and atheroprotective effects, particularly in the early phases of atherosclerosis. Consequently, blocking PD-1 as part of cancer therapy may increase cardiovascular risk and potentially accelerate atherosclerosis progression [57].

Clinical evidence supports these concerns. A study involving 2842 cancer patients undergoing ICI therapy found that those treated with ICIs had a three-fold higher risk of experiencing atherosclerosis-mediated cardiac events [58]. Additionally, ICIs have been associated with an increased prevalence of vulnerable plaques characterized by reduced fibrous caps, predisposing patients to plaque rupture and thrombotic events such as myocardial infarction and ischemic stroke [59].

Autopsy findings further underscore the pro-atherogenic effects of ICIs. In cancer patients treated with ICIs, heightened inflammatory cell infiltration has been observed in coronary plaques, highlighting the role of these therapies in promoting plaque instability and cardiovascular complications [60].

Table 1 systematically presents the main pharmacological groups of anticancer chemotherapeutic agents and their association with ASCVD.

### 4.11. Confounding Factors

The assessment of cardiovascular risks associated with anticancer therapies is inherently complicated by the presence of multiple confounding factors. These include pre-existing cardiovascular risk profiles such as age, sex, hypertension, diabetes mellitus, dyslipidemia, and lifestyle factors, including smoking, physical inactivity, and dietary habits [61,62,63]. Cancer itself, as a pro-inflammatory and hypercoagulable state, introduces additional complexity by independently contributing to atherosclerotic risk [64]. This overlap makes it particularly challenging to disentangle the direct effects of anticancer therapies from the baseline cardiovascular risks conferred by the disease.

Comorbidities, treatment duration, and the intensity of chemotherapy regimens further influence cardiovascular outcomes. Individual variability in drug metabolism and response also plays a pivotal role in modulating risk. For example, the co-administration of corticosteroids, often used to mitigate chemotherapy-related side effects, can exacerbate dyslipidemia and hyperglycemia, thereby compounding the overall cardiovascular burden. Similarly, supportive therapies such as erythropoiesis-stimulating agents and growth factors, while essential for managing treatment-induced anemia or neutropenia, may inadvertently impact vascular health by promoting thrombosis or endothelial dysfunction.

These interrelated factors highlight the need for a comprehensive, individualized approach to cardiovascular risk assessment and management in cancer patients. Identifying and addressing confounding variables is essential to accurately evaluate the cardiovascular impact of anticancer therapies and develop targeted mitigation strategies.

## 5. Discussion

Chemotherapeutic agent-induced ASCVD is a multifactorial condition arising from the interplay between direct pharmacological effects and patient-specific baseline cardiovascular risk factors. Mechanisms such as endothelial dysfunction, oxidative stress, and systemic inflammation underscore the complexity of balancing effective cancer treatment with maintaining cardiovascular health [19,65]. For instance, anthracyclines induce mitochondrial dysfunction and oxidative stress, while ICIs exacerbate inflammatory pathways, destabilizing atherosclerotic plaques and elevating ASCVD risk through distinct mechanisms [15,16,26].

Early identification of chemotherapy-induced ASCVD risk factors is pivotal for improving patient outcomes. Comprehensive cardiovascular risk stratification before treatment, incorporating traditional risk factors and cancer-specific considerations is essential. The 2022 ESC guidelines on cardio-oncology emphasize systematic baseline assessments, including advanced imaging techniques where feasible, to detect subclinical atherosclerosis [26].

Pre-treatment risk assessment involves several key components, with patient history and demographics playing a significant role in determining cardiovascular risk. Factors such as age, sex, and ethnicity are particularly influential. Older patients, postmenopausal women, and certain ethnic groups, including South Asians and African Americans, have been identified as having an elevated risk of cardiovascular complications [66,67]. For instance, studies have demonstrated that long-term treatment outcomes in breast cancer survivors are closely linked to atherosclerotic changes [68]. Additionally, pre-existing cardiovascular conditions, such as coronary artery disease, hypertension, diabetes, or hyperlipidemia [69], heighten patient vulnerability, emphasizing the importance of their early identification.

Another critical consideration is the recognition of therapy-related risk factors. High-risk cancer therapies, such as anthracyclines, which generate high levels of ROS and induce direct endothelial damage [22], platinum-based agents associated with vascular stiffness [70], and VEGF inhibitors that impair microvascular function [71], must be carefully evaluated for their cardiovascular implications.

Lifestyle and behavioral factors also significantly impact cardiovascular risk. Smoking, physical inactivity, and poor nutrition exacerbate risk, highlighting the need for preventive behavioral interventions [72]. These modifiable factors underscore the importance of integrating lifestyle counseling into pre-treatment care plans to mitigate adverse outcomes.

In addition, the gut microbiome plays a pivotal role in cardiovascular risk by producing trimethylamine (TMA) from dietary phosphatidylcholine, which is converted in the liver to trimethylamine-N-oxide (TMAO), a metabolite linked to atherosclerosis and systemic inflammation. Chemotherapy-induced gut dysbiosis amplifies these effects by increasing TMA/TMAO production and disrupting microbial communities critical for vascular health. Dysbiosis also reduces short-chain fatty acids (SCFAs), such as acetate, propionate, and butyrate, which regulate inflammation and lipid metabolism. Reduced SCFAs contribute to gut permeability, allowing microbial components like lipopolysaccharides (LPSs) into circulation, triggering systemic inflammation and atherogenesis [73].

Chemotherapy exacerbates these risks by altering gut microbial composition, disrupting SCFA production, and activating pro-inflammatory pathways. These changes promote endothelial dysfunction, lipid accumulation, and vascular instability, illustrating the complex interplay between gut health and cardiovascular risk in cancer patients [73].

Moreover, anticancer therapies have been shown to impact gut microbial diversity and composition, further exacerbating dysbiosis. For example, certain chemotherapeutic agents induce microbial imbalances that favor pro-inflammatory pathways, whereas others may disrupt SCFA production or alter bile acid metabolism, indirectly promoting endothelial dysfunction and lipid accumulation [73].

Physical activity and diet help mitigate these effects. Exercise improves lipid profiles, reduces inflammation, and counters chemotherapy-induced oxidative stress and metabolic derangements. A nutrient-rich diet high in fiber, omega-3 fatty acids, and antioxidants supports vascular health, reduces TMAO levels, and promotes gut microbiota balance, further protecting against cardiovascular complications [74].

Comprehensive laboratory and imaging evaluations further enhance pre-treatment risk stratification. Tests such as lipid profiles, cardiac biomarkers (e.g., troponin and CRP), and imaging techniques like carotid intima–media thickness measurement and coronary artery calcium scoring provide invaluable insights into subclinical ASCVD [26]. These assessments enable clinicians to identify at-risk patients before symptoms develop.

Pharmacological optimization prior to treatment has also shown great promise in reducing cardiovascular risk. Medications such as statins, ACE inhibitors, and angiotensin receptor blockers (ARBs) are invaluable tools in mitigating cardiovascular complications [75,76,77]. Personalized management of hypertension and hyperlipidemia tailored to the individual patient’s needs ensures effective risk reduction and enhances the safety of cancer therapy [11,15,25,26].

The adoption of a multidisciplinary approach is essential for the effective management of ASCVD induced by cancer therapy. A collaborative strategy that integrates oncology and cardiology can address the complexities of chemotherapy-induced cardiovascular complications [78,79,80]. Establishing dedicated cardio-oncology units within healthcare centers, including resource-limited settings, bridges the gap between these specialties [81]. These units enable context-specific interventions to tackle regional challenges while optimizing patient outcomes [26].

Lipid-lowering therapy, particularly with statins, remains the cornerstone of dyslipidemia management in cancer patients [82]. Statins not only provide cardiovascular protection but also offer potential anticancer benefits. For example, in anthracycline-based treatments, statins have been shown to reduce the risk of damage to central blood vessels during therapy [82,83]. In resource-limited settings, prioritizing generic and cost-effective statins is critical to ensure widespread access. The dual role of lipid-lowering therapy in reducing ASCVD risk and improving cancer survival underscores its significance in cardio-oncologic care [15,41].

Aspirin is another commonly used agent in this population. While it has demonstrated efficacy in reducing adverse cardiovascular events associated with chemotherapeutic drugs, its use must be carefully evaluated due to the increased risk of bleeding [84]. Additionally, dexrazoxane, an iron-chelating agent, mitigates the cardiotoxic effects of doxorubicin by preventing free radical formation [85]. Other agents, such as TNF inhibitors, have shown potential in preventing coronary atherosclerosis by targeting inflammatory pathways [86].

Lifestyle modifications play a critical role in mitigating the risk of cancer therapy-induced ASCVD [87]. Behavioral changes, including dietary improvements, regular physical activity, and smoking cessation, are integral to cardiovascular risk reduction. Public health initiatives targeting these modifiable risk factors are particularly crucial in underserved regions, complementing individual-level strategies to improve overall outcomes [11,15,26].

Continuous surveillance of cardiovascular events during and after chemotherapy is imperative [88]. Long-term follow-up ensures continuity of care, particularly in underserved areas, by facilitating the timely detection of complications. Early intervention during follow-up reduces long-term morbidity and enhances patient outcomes [26].

## 6. Conclusions

Chemotherapy-induced CVAD is a complex condition resulting from the interplay between the direct pharmacological effects of anticancer agents and patient-specific cardiovascular risk factors. Key mechanisms driving CVAD—such as endothelial dysfunction, oxidative stress, systemic inflammation, and lipid metabolism alterations—highlight the necessity of a proactive and integrated approach to management.

Comprehensive pre-treatment cardiovascular risk assessment is crucial and should consider demographic, lifestyle, and therapy-specific factors alongside advanced diagnostic tools to detect subclinical disease. Early identification of at-risk patients allows for tailored strategies to mitigate complications.

Pharmacological interventions, including statins and ACE inhibitors, alongside lifestyle modifications, play a pivotal role in reducing CVAD risk. Multidisciplinary collaboration within cardio-oncology units ensures optimal care coordination, effectively addressing the multifaceted cardiovascular complications of cancer therapy.

In resource-limited settings, prioritizing affordable solutions, such as generic statins and public health initiatives targeting modifiable risk factors like smoking and inactivity, can improve accessibility and health outcomes.

Continuous cardiovascular monitoring during and after treatment is essential for early detection of complications and timely intervention. These measures collectively reduce the burden of CVAD, enhance survival, and improve the long-term quality of life for cancer patients across diverse care settings.

## Figures and Tables

**Table 1:** Summary of evidence of anticancer drugs and their mechanisms related to ASCVD effects.

Anticancer Agent Class	Mechanisms of Action	Effects on ASCVD	Clinical Implications
Anthracyclines	Inhibit topoisomerase II, induce mitochondrial dysfunction, generate ROS	Impair endothelial function, promote oxidative stress, disrupt cholesterol efflux, and elevate ApoB levels	Promote plaque formation and instability by reducing HMGCR activity despite elevated LDL levels
Taxanes	Stabilize GDP-bound tubulin, disrupting microtubule function	Alter lipid metabolism: increase HMGCR activity, reduce ApoB activity, and lower LDL receptor expression	Associated with dyslipidemia and proteomic changes in lipid metabolic pathways
VEGF Inhibitors	Block tumor angiogenesis, impair endothelial homeostasis	Reduce NO and PGI2 levels; promote vasoconstriction, platelet aggregation, microvascular thrombosis	Accelerate plaque progression, increase hypertension risk, and raise overall ASCVD incidence
Anti-Metabolites	Incorporated into DNA/RNA, disrupting cell proliferation	Increase ROS, pro-inflammatory cytokines, adhesion molecules, and endothelial stress	Contribute to endothelial injury, plaque vulnerability, and coronary vasospasm in some cases
mTOR Inhibitors	Inhibit mTOR signaling, affecting cell proliferation and metabolism	Downregulate lipogenic enzymes and LDL receptors; impair autophagy and endothelial repair	Induce dyslipidemia (elevated LDL/TG) and potentially impact ASCVD outcomes, though evidence is limited
Hormonal Therapies	Modulate estrogen/testosterone via SERMs, ADT, or AIs	Affect cholesterol synthesis and lipid transport; SERMs modulate lipid profiles, while AIs increase risk	ADT raises ASCVD risk (~20%), while SERMs offer some lipid protection; AIs increase dyslipidemia and CVD events
Tyrosine Kinase Inhibitors (TKIs)	Inhibit kinases involved in endothelial repair and NO production	Increase ROS, lipid peroxidation, and impair endothelial function	Imatinib reduces cholesterol and atherosclerosis; newer TKIs show mixed effects on lipids
Alkylating Agents	Cross-link DNA and RNA, halting cell division	High doses induce dyslipidemia in animals; dyslipidemia association unclear in humans	High doses induce dyslipidemia in animals; low-dose cyclophosphamide reduces atherosclerosis; human data limited
Platinum-Based Agents	Cross-link DNA, disrupting transcription	Promote endothelial dysfunction, increase cytokines, and induce fibrinogen	Associated with thrombotic and inflammatory states; little data on cholesterol or lipid changes in humans
Immune Checkpoint Inhibitors (ICIs)	Block PD-1 and CTLA4, enhancing T-cell activity	Exacerbate plaque inflammation, increase cytokines, promote vulnerable plaques	Linked to a 3-fold higher ASCVD risk, including plaque rupture and thrombotic complications

## Data Availability

Not applicable.

## Abbreviations

The following abbreviations are used in this manuscript:

ASCVD	atherosclerovascular disease
LDL	low-density lipoprotein
HDL	high-density lipoprotein
oxLDL	oxidized low-density lipoprotein
VEGF	vascular endothelial growth factor
TNF-α	tumor necrosis factor-alpha
IL	interleukin
IFN-γ	interferon-gamma
SASP	senescence-associated secretory phenotype
ROS	reactive oxygen species
DDR	DNA damage response
NO	nitric oxide
PGI2	prostacyclin
TS3	Type III Secretion System
Hfq	Host Factor for RNA Binding
mTOR	mammalian target of rapamycin
HMGCR	3-hydroxy-3-methylglutaryl-coenzyme a reductase
LXR-α	liver X receptor-alpha
PPAR-γ	peroxisome proliferator-activated receptor-gamma
ABCA1	ATP-binding cassette transporter A1
ApoB	apolipoprotein B
GDP	Guanosine Diphosphate
5-FU	5-fluorouracil
TG	triglyceride
ICAM-1	Intercellular Adhesion Molecule 1
VCAM-1	Vascular Cell Adhesion Molecule 1
ADT	androgen deprivation therapy
SERMs	selective estrogen receptor modulators
AIs	aromatase inhibitors
TKIs	tyrosine kinase inhibitors
PD-1	Programmed Death-1
CTLA4	Cytotoxic T-Lymphocyte-Associated Protein 4
CVD	cardiovascular disease
CRP	C-reactive protein
ARB	angiotensin receptor blocker
CVAD	cardiovascular adverse disease
TMA	trimethylamine
TMAO	trimethylamine-N-oxide
SCFAs	short-chain fatty acids
